# Induced and Evoked Brain Activation Related to the Processing of Onomatopoetic Verbs

**DOI:** 10.3390/brainsci12040481

**Published:** 2022-04-06

**Authors:** Dorian Röders, Anne Klepp, Alfons Schnitzler, Katja Biermann-Ruben, Valentina Niccolai

**Affiliations:** 1 Institute of Clinical Neuroscience and Medical Psychology, Medical Faculty, Heinrich-Heine University, 40225 Duesseldorf, Germany; anne.klepp@fernuni-hagen.de (A.K.); alfons.schnitzler@uni-duesseldorf.de (A.S.); katja.biermann-ruben@med.uni-duesseldorf.de (K.B.-R.); valentina.niccolai@med.uni-duesseldorf.de (V.N.); 2 Neural Basis of Learning Lab, Institute for Cognitive Neuroscience, Faculty of Psychology, Ruhr University, 44801 Bochum, Germany

**Keywords:** onomatopoeia, verbs, beta, alpha, ERF, MEG

## Abstract

Grounded cognition theory postulates that cognitive processes related to motor or sensory content are processed by brain networks involved in motor execution and perception, respectively. Processing words with auditory features was shown to activate the auditory cortex. Our study aimed at determining whether onomatopoetic verbs (e.g., “tröpfeln”—to dripple), whose articulation reproduces the sound of respective actions, engage the auditory cortex more than non-onomatopoetic verbs. Alpha and beta brain frequencies as well as evoked-related fields (ERFs) were targeted as potential neurophysiological correlates of this linguistic auditory quality. Twenty participants were measured with magnetoencephalography (MEG) while semantically processing visually presented onomatopoetic and non-onomatopoetic German verbs. While a descriptively stronger left temporal alpha desynchronization for onomatopoetic verbs did not reach statistical significance, a larger ERF for onomatopoetic verbs emerged at about 240 ms in the centro-parietal area. Findings suggest increased cortical activation related to onomatopoeias in linguistically relevant areas.

## 1. Introduction

The theory of grounded cognition proposes that cognition is dependent on the brain’s modal systems for perception, action and introspection [1]. This theory postulates that the sensory and motor brain areas are activated not only during perception or action, but also by cognitive processes such as understanding words related to these modalities. Some studies show that this is true, for example, for the motor area: reading hand- and foot-related action words activate areas belonging to the motor cortex and responsible for hand and foot movements, respectively [2,3,4,5,6,7,8]. Analogously, words implying acoustic features were shown to activate, beyond other areas, part of the same temporal brain area also recruited during sound perception [9]. Behavioural findings showed that reading auditory-related verbs improved the detection of subsequent hardly audible sounds in participants with high lexical decision performance [10]. So far, there is a lack of research about such cognitive simulation processes involving the auditory system during word processing and even less studies focussed on neural oscillations in this context. The power of brain oscillations can be used as an index of neural activation level. While synchronized beta oscillations (12–25 Hz) have been proposed to maintain the current cognitive or sensorimotor state, desynchronized beta oscillations have been interpreted also as local cortical activation, for example, related to movements or to auditory processing [11]. Synchronization of the alpha frequency (8–12 Hz) is viewed as an idle state of the brain [12] while, e.g., alpha (8–12 Hz) desynchronization in the auditory cortex has been shown to accompany auditory stimulation [13]. Within the framework of the grounded cognition theory, it was found that visually presented words describing loud actions induced stronger beta frequency desynchronization in the left auditory cortex compared to words describing quiet actions [14]. 

Onomatopoetic words are especially interesting in this context as they tend to acoustically reproduce the sound (and sometimes the shape or even other semantic qualities) of the object or action they refer to [15,16]. In earlier studies, onomatopoetic words were shown to be accompanied by stronger activation in those areas that are usually activated by the related real-sound stimuli: for example, animal sound-related onomatopoetic words (e.g., the Japanese word “wanwan” indicating the dog’s barking) activated areas responsible for the perception of non-verbal sounds [17,18,19,20,21,22]. However, these studies exclusively focussed on interjections, that is, words that only imitate a sound (e.g., “kikeriki” for a rooster call); these, however, are neither verbs, nor nouns, nor adjectives. Profiting from the strong onomatopoetic quality of interjections, most studies so far compared these to other non-onomatopoetic word classes to determine the effect of onomatopoeias on brain and behaviour [15,17,18,19,20,22,23,24,25,26]. Auditorily presented onomatopoetic interjections were shown to activate the auditory cortex and, specifically, the bilateral middle and anterior superior temporal sulcus (STS) more strongly than non-onomatopoetic nouns with the same reading frequency, auditory familiarity and auditory imageability [22]. Similarly, activation of the right posterior superior temporal sulcus (pSTS) following onomatopoetic word presentation was also found in another study [24]. Whereas these studies hint at a peculiar effect of onomatopoetic words, the comparison of interjections with non-onomatopoetic words belonging to different grammatical classes is problematic. Since the grammatical class of the word stimuli influences the localization and strength of brain activation as well [23,27], comparing interjections with verbs might result in effects going beyond onomatopoeias. 

Few electroencephalography (EEG) studies applied onomatopoetic words instead of interjections; auditorily presented onomatopoetic adverbs (e.g., the Japanese “gatagata” for “rattling”) were found to elicit a larger late-positive sustained complex at about 400–800 ms than control adverbs, thus reflecting increased post-lexical processing [23]. In another study, processing visually presented onomatopoetic verbs resulted in a less negative-going N400 component and late-positive deflection compared to non-onomatopoetic control verbs [28]. The authors interpreted their findings as onomatopoeias being easier to process. However, results from an additional behavioural task in Peeters’ study showed that participants were not faster in differentiating onomatopoetic verbs from non-words than differentiating non-onomatopoetic verbs from non-words. This behavioural finding thus does not support the notion of the easier processing of onomatopoeias. Altogether, the literature is scarce and inconsistent, to some extent. 

In the current MEG study, we aimed at determining the oscillatory as well as evoked neurophysiological activation related to onomatopoeias by comparing German onomatopoetic verbs (e.g., “brummen”—to hum) to non-onomatopoetic verbs matched for frequency, length and implied loudness. The latter was meant at controlling for a dimension of acoustic relevance. We focussed on the temporal cortical areas, because of their role in auditory processing and on the base of the literature on onomatopoeias [22,24]. For the aim of the current analyses, we selected the MEG channels resulting from a previous auditory localizer paradigm from our work group [14]. Here, onomatopoetic verbs were expected to induce larger alpha and beta frequency desynchronization in comparison to non-onomatopoetic verbs as a consequence of the increased engagement of the auditory cortex. Regarding evoked fields, we expected an overall facilitated linguistic processing of onomatopoetic verbs to reflect onto a lower amplitude than non-onomatopoetic verbs [28].

## 2. Materials and Methods

### 2.1. Participants

Twenty (10 females, 10 males, average age = 28.9 ± 6.9) right-handed (laterality Quotient = 94.2 ± 9.6 [29]), monolingual, German native speakers with no formal training in linguistics participated in the MEG study. Subjects had normal or corrected to normal vision, had no neurological or psychiatric disorder and were not using psychotropic medications. Left-handed people were excluded, as right- and left-handed participants show different cortical language dominance [30]. Linguists were excluded to avoid focussing on specific linguistic aspects of the presented words and an implicit advantage compared to non-linguists. Non-native speakers were not included in the study because different brain language areas have been found to be activated by foreign versus native [31]. Even if onomatopoetic foreign words may be intuitively easier to understand for non-native speakers than non-onomatopoetic ones [32], the related cortical activation might still be qualitatively different from that of native speakers. Participants were kept unaware of the purpose of the study to prevent interference with cognitive processes. After the completion of the experiment, participants were asked to guess the study purpose, and they were debriefed. 

### 2.2. Stimuli

An initial list of 136 German verbs describing actions related to sounds was created, and they were initially pre-grouped in onomatopoetic and not onomatopoetic words. These verbs were then evaluated by means of an online questionnaire (https://soscisurvey.de, 15 August 2019) by German native speakers. Only fully completed questionnaires were used (n = 38, 20 females, 18 males, average age = 32.7 ± 14.5). Participants were asked to rate each verb regarding familiarity, onomatopoeias, sound source (human vs. environmental sounds) and loudness on a 1–4 Likert scale. To ensure that the participants had a sufficient understanding of the concept of onomatopoeias, they were told that an onomatopoeia describes how much the pronunciation of the verb imitates the sound associated with the implied action. Participant were then asked to “please rate how much the pronunciation of the following word imitates the sounds associated with them”. They were also given example words, such as “to excavate” (baggern) as an example for a non-onomatopoetic word and “to hiss” (fauchen) as an example for a very onomatopoetic word. These participants were not included in the MEG study to prevent a priori knowledge of the stimuli. Items were presented in a random order to avoid systematic confounding effects (e.g., tiredness). Based on the results of the questionnaire, 49 words with the highest (3.1–2.5) and 56 with the lowest (2.2–1.3) onomatopoeia rating values were preliminarily assigned to the respective conditions. The two groups of verbs were further matched for length (*p* = 0.407), word frequency (*p* = 0.105), sound source (*p* = 0.736) and loudness rating values (*p* = 0.189). The resulting onomatopoetic and non-onomatopoetic words differed significantly for onomatopoeias (average 2.8 vs. 1.9; v = 0; *p* < 0.001). The matching procedure resulted in 34 verbs for each condition. Non-onomatopoetic verbs were significantly more familiar than onomatopoetic words (*p* = 0.020). Since this could not be avoided without drastically shrinking the number of words per category, we opted for these verbs. The length and word frequency values were tested for significant differences with a Student’s *t*-test. All other values were tested with a Wilcoxon test. All the above-mentioned tests were run with R version 3.5.2 (https://www.r-project.org/, 28 January 2019). The matching process was performed in a semiautomatic way with the program Match [33]. Verbs used in the MEG study (34 per group) are presented in Table S1.

**Figure 1:**
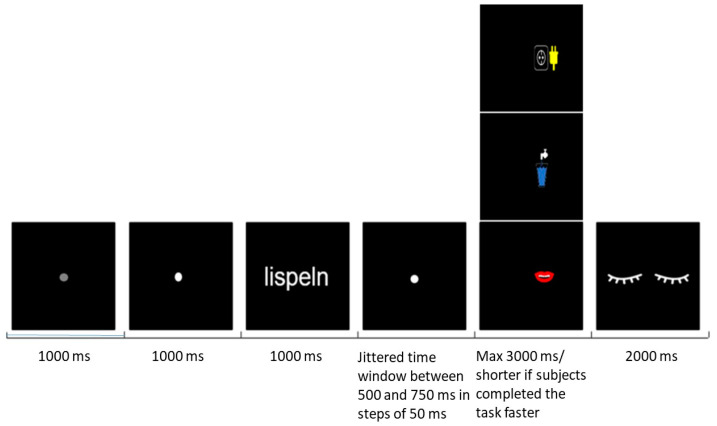
Experimental design.

During the MEG measurement, the following task and trial design was applied (Figure 1): a grey fixation point was presented for 1 s, followed by a white fixation point lasting 1 s and indicating the upcoming verb. The word then appeared for 1 s, followed again by a fixation point lasting 500 to 750 ms with a jittered interval in steps of 50 ms; a jitter was used to prevent response automatization. The prompt displayed one out of three possible symbols representing a glass of water, a mouth and an electric outlet with a plug (Figure 1). In order to induce the semantic processing of word stimuli and to keep the participant unaware of the study conditions and purpose, each symbol was associated with one of the following questions, respectively:

1. Has the process implied by the verb anything to do with liquids?
2. Is the process implied by the verb performed with the mouth?
3. Is the process implied by the verb performed with an electric tool?

The prompt was presented either on the right or on the left side of the screen. The participants were required to respond “yes” to the prompt by lifting the index finger of the hand positioned on the same side as the presented symbol and “no” by lifting the index finger of the opposite hand. Left- and right-hand responses were balanced pseudo-randomly in order to trigger 50% right- and 50% left-hand responses. To reduce eye movement-related artefacts, participants were asked to avoid blinking until the end of the trial, when an eye symbol lasting 2 s indicated to blink. All 68 verbs were presented 3 times across 3 blocks. Each word was always followed by one of the questions above (Table S1). Blocks were separated by pauses as long as needed by the participant. Words were presented in a randomized order within each block. The measurement lasted about 35 min, depending on participants’ reaction and pause time. 

### 2.3. Procedures

After signing informed consent and data privacy forms, participants filled out the Edinburgh Handedness Inventory [29]. They were asked to remove metal belongings, and if needed, were offered metal-free cotton clothes as well as individually calibrated metal-free glasses with corrective lenses. For electrooculography (EOG), four electrodes were placed around the eyes: one above and one under the left eye for vertical EOG and two at about 1 cm from the left and the right eye for horizontal EOG. These bipolar electrodes were used to detect eye movements and blinks. Four coils were placed on the forehead and behind the ears. The positions of the coils were digitized (Polhemus Isotrak) for later estimation of the head position during MEG measurements. During the MEG measurement, the participants were seated comfortably with their hands resting on two pads and their index fingers on two photoelectric switches. Instructions and word stimuli were projected onto a screen in front of the participant. After three demonstration trials, participants performed three practice trials that could be repeated, if needed, before starting the measurement.

### 2.4. Data Acquisition and Analysis

Neuromagnetic brain activity was recorded with a 306-channel MEG system (Elekta Neuromag, Helsinki, Finland). The channels consisted of 102 magnetometers and 204 orthogonal planar gradiometers. MEG data were digitized at 1000 Hz, bandpass filtered from 0.03 to 330 Hz online and stored on a computer hard disk.

MEG data were analysed with Matlab R2017b and fieldtrip toolbox [34]. Behavioural data analysis was run with R version 3.5.2 [35].

### 2.5. Meg Data Pre-Processing

Epochs were cut from the continuous data and included the time window between 1 s before word onset and 1 s after word onset. Only correct trials entered the analysis. Trials with answers at wrong time points or double answers were excluded from analyses. Semiautomatic jump and muscle artifact rejection was applied to the selected epochs. A notch filter was used to filter out the frequencies 49–51, 99–101 and 149–151 Hz. A high-pass filter of 2 Hz and a padding of 5 s were used as well. Heart and eye-related artifacts were removed via independent component analysis [36]: this resulted in the elimination of, on average, 2.6 components per subject. Noisy or faulty channels were repaired by interpolating data from neighbouring channels. An average of 6 surrounding gradiometers of the same type were used for each faulty channel. Trials were visually inspected for residual artifacts and then assigned to the two conditions.

### 2.6. Time–Frequency Representations and Event-Related Field Analysis

Time–frequency representations were calculated by using a fast Fourier transformation. An adaptive sliding time window including 5 cycles was shifted in steps of 50 ms from −1 s to 1 s after word onset. Data were padded up to 5 s. A single Hanning taper was applied, and power was estimated in steps of 1 Hz between 2 and 40 Hz. The time–frequency analysis was performed separately for horizontal and vertical planar gradiometers, and the pairs of planar gradiometers were combined afterwards. The time from 600 ms before word onset to 100 ms before word onset served as a baseline.

For the computation of ERFs, data were filtered with a low pass filter of 30 Hz. For each subject episodes from −1 s to 1 s after word onset were averaged; the time interval from −200 ms to word onset (=0 ms) served as the baseline. Horizontal and vertical planar gradiometers were combined. 

### 2.7. Statistics

Difference in reaction time between word conditions and question types were tested with an ANOVA.

Considering the multidimensionality of MEG data, for the frequency and ERFs analysis, a procedure that effectively corrects for multiple comparisons, a non-parametric randomisation test, was used [37]. With regard to frequency analysis, the contrast between onomatopoetic and non-onomatopoetic words was run in the alpha and beta range (8–25 Hz), across the time window between 0 and 1 s after word onset (no average over time) and on the average of the activity of 9 left hemispheric temporal channels (Figure S1) that were selected on the base of results of a previous MEG localizer study targeting the auditory cortex [14]. A one-sided *t*-test for dependent samples was used. T-values of the time–frequency samples passing the significance threshold (*p* < 0.05) were selected and clustered with adjacent time and frequency bins. A cluster-level statistic was then calculated by taking the sum of the *t*-values of the samples within every cluster. A non-parametric permutation test, which consisted in computing 1000 random sets of permutations between the two conditions, was used to obtain a distribution of the cluster statistic; the significance level was set to *p* < 0.05. 

The same procedure was applied to the statistical analysis of ERFs for the contrast between the onomatopoetic and non-onomatopoetic verb condition. The analysis included all channels. Considering the evidence for early semantic processes [38,39,40,41], we targeted the time window between 100 and 300 ms after word onset to detect semantically related components. Group differences in ERFs amplitude were also tested with a one-sided *t*-test, as onomatopoetic verbs were expected to elicit larger amplitudes. 

## 3. Results

### 3.1. Behavioural Results

The reaction time for onomatopoetic verbs (on average, 741 ms ± 266 ms) was significantly shorter than for non-onomatopoetic words (on average 748 ms ± 326 ms; (*p* < 0.001)). The type of question did not have a significant effect on reaction times (*p* = 0.465). Missing responses were, on average, 0.3% per subject. 

Incorrect responses occurred in an average of 6.4% of trials per subject. No participant thus exceeded the 15% error cut-off, at which the participant’s data would have been discarded: this suggests that the task was not too difficult for the participants. As no participant was able to correctly guess the purpose of the study, correct trials of all subjects entered the analyses.

### 3.2. Time–Frequency Representations

**Figure 2:**
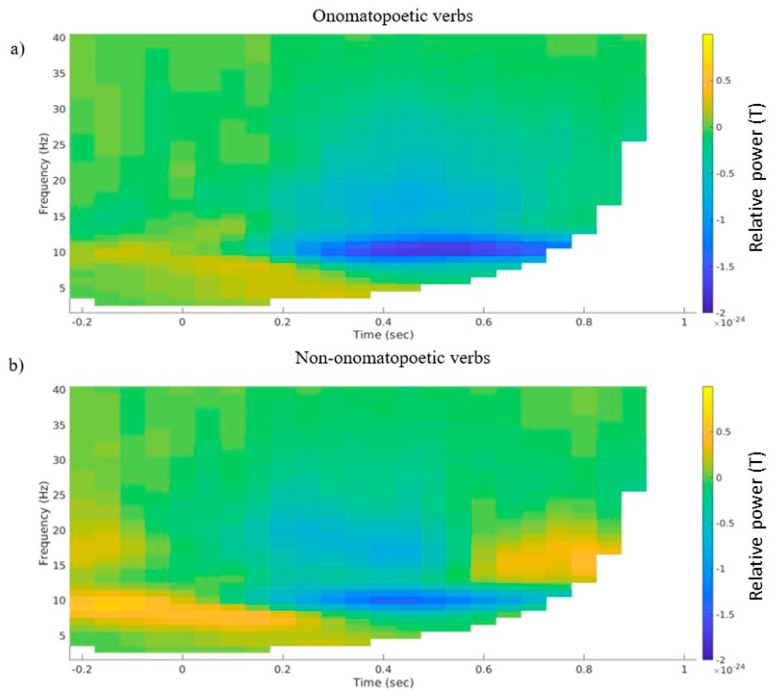

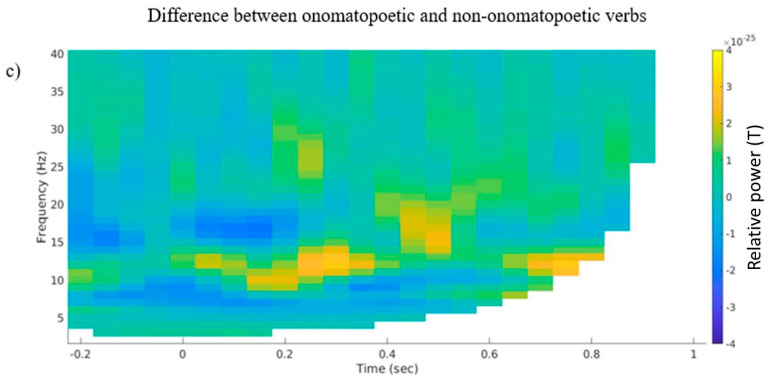
(**a**) Grand average time–frequency representations of the averaged selected left temporal channels for (**a**) the onomatopoetic verb condition, (**b**) the non-onomatopoetic verb condition and (**c**) the difference between onomatopoetic and non-onomatopoetic verb condition.

A statistical analysis of alpha and beta power on the nine selected channels yielded no significant result; no negative cluster emerged. However, on a descriptive level, differences in alpha and beta power emerged mainly in the left temporal channel selection (Figure 2). Here, a desynchronization in both frequency ranges was visible starting at about 200 ms after word onset, both in the onomatopoetic and the non-onomatopoetic verb condition (Figure 2a,b). The onomatopoetic condition showed a slightly increased alpha desynchronization, between 400 and 600 ms, and beta desynchronization between 0 and 200 ms as well as at about 700 ms after stimulus onset (Figure 2c). A descriptively stronger synchronization in the alpha range between 200–400 ms and in the beta range around 400–500 ms was also visible.

### 3.3. Event-Related Fields

**Figure 3:**
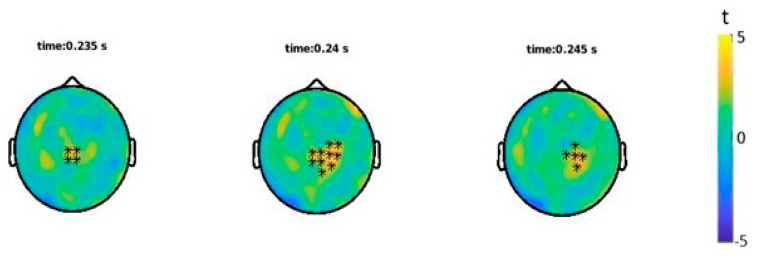
Statistical results of ERFs analysis: channels showing a significant effect (*) in the shown time interval.

**Figure 4:**
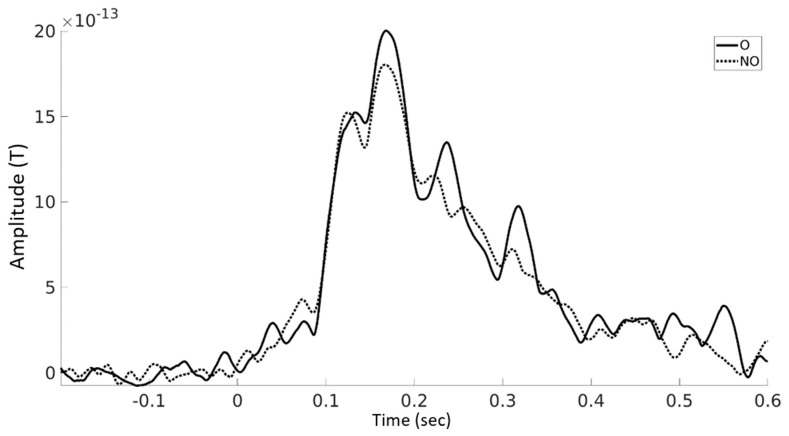
Averaged ERF amplitudes for onomatopoetic verbs and non-onomatopoetic verbs until 600 ms after word onset across all channels showing a significant effect (see Figure 3).

ERFs analyses showed a statistically significant difference (*p* = 0.033) between the onomatopoetic and non-onomatopoetic condition around 240 ms after word onset with larger amplitudes for onomatopoetic words (Figure 3 and Figure 4). The difference emerged on centro-parietal channels and then shifted to slightly right lateralised sites. 

## 4. Discussion

Accuracy results showed that the participants did semantically process the words in the given time. Reaction time was shorter for onomatopoetic in comparison to non-onomatopoetic verbs, even though familiarity was significantly lower for onomatopoetic verbs and should thus increase reaction time. This suggests that onomatopoetic words are easier to understand, possibly depending on the non-arbitrary link between the word sound and its meaning. In contrast, the oscillatory and the ERFs patterns of activation seem to indicate a more effortful processing of onomatopoetic verbs. In a behavioural study also applying auditory onomatopoetic versus control verbs, no difference in reaction time emerged [29]. Since in that study the task consisted in distinguishing words from pseudo-words, a possible difference in processing ease was suggested to be obscured by task-related decision making and motor processes, which might require more time than the lexical processing. This suggests that semantic versus lexical processing, which reflects the depth of linguistic processing, may be responsible for the emergence of behavioural effects. A role of the depth of semantic processes in the emergence of embodiment effects was indeed shown in a previous study of our group, where semantic discrimination impacted the modulation of verb processing as induced by electrical stimulation [42]. However, differences in reaction time in the current study should be interpreted with caution, since our task was not a simple reaction time task as in Peeters’ study. 

Both onomatopoetic and non-onomatopoetic words showed alpha and beta desynchronization starting at about 200 ms after word onset in the left temporal lobe: this result adds evidence to the role of alpha and beta desynchronization as a marker of semantic processing. Although not reaching statistical significance, the slightly decreased alpha and beta power accompanying onomatopoetic verbs in the selected left temporal channels suggests that this linguistically predominant hemisphere might be sensitive to onomatopoeias. Similarly, increased left temporal beta desynchronization accompanies words implying loud vs. quiet actions [14]. On the base of these results, onomatopoetic verbs were expected to cause a stronger recruitment of the auditory cortex due to their linking function between semantics and phonetics. The synchronization visible in the alpha band around 200–400 ms and in the low beta band around 400–500 ms is more difficult to explain. It was not expected to be a marker of increased cortical engagement in the context of embodied semantics, but considering its latency, we cannot exclude a relation to particular semantic diverging aspects between the two conditions. Beta oscillations in particular are also related to complex linguistic sub-processes, to expectancy violation and attention as well as to working memory [43]. Whether familiarity, which was rated higher for non-onomatopoetic words, might be responsible for this effect, remains unclear. One limitation of the current study is that additional word-related parameters such as imageability, age of acquisition and emotional valence were not rated and controlled for. Possibly, even more linguistic parameters might affect ERF amplitude or brain oscillations; this needs to be further determined with studies specifically designed for this purpose. To our knowledge, this is the first study addressing oscillatory correlates of onomatopoetic versus non-onomatopoetic verb processing, and we cannot report a significant difference in brain oscillations. Previous studies using interjections compared to verbs point to stronger onomatopoetic qualities of these words and to a stronger activation of the auditory cortex. This might be an explanation as to why our word stimuli with weaker onomatopoetic qualities did not engage the auditory cortex as much as previously used stimuli. Although previous studies have matched interjections and control words for imageability, familiarity and age of acquisition [24], the two conditions included different grammatical categories. The use of verbs in the present study allowed a better control of grammatical aspects as well as of other related parameters such as length, word frequency and loudness. By controlling for linguistically confounding effects, we improved the comparability between conditions. Increasing semantic task difficulty might help determining a neurophysiological effect of this subtle semantic quality that is the onomatopoeia. It is worth noting that half of the words used in our study described events that were not primarily associated with human actions, but more with environmental events (e.g., “surren”—to whir, “zischen”—to hiss and “plaetschern”—to platter). Since environmental events and human actions were balanced between conditions, and the sound source should not have affected results. Still, it might have impaired simulation processes by moving the attentional focus to an extra-personal space. Verbs related to actions in which participants can envision themselves as actors are likely to induce stronger simulation. 

ERF analysis showed a significant effect emerging at about 240 ms after word onset in the centro-parietal sensors, suggesting increased cortical activation related to onomatopoetic verbs. This hints at a more effortful processing of onomatopoetic verbs: as proposed in a previous study [28], onomatopoetic verbs have a duality of lexical and sound components, which creates a processing conflict. Peeters [28] argued that this is compensated by an easier understanding due to the link between the word content and the way the word is pronounced. While this was not confirmed by the behavioral results, the current findings point in that direction and show faster reaction times following onomatopoetic verbs despite the jittered time interval between the word and prompt onset. 

The current results are in line with those of EEG studies showing differences in the ERPs when comparing acoustically presented onomatopoetic verbs to control verbs [28] as well as comparing visually presented ideophones (which are regarded as either very similar to or as the same as interjections) to control adverbs [23]. Peeters [28] found a significant amplitude decrease of the N2 component, a less negative-going N400 and a late-positive deflection compared to the control words distributed over all cortical areas. Lockwood and Tuomainen [23] found ERP effects at roughly the same time points as Peeters [28], but with a more negative going N400 for ideophones than for control words. We found significant differences in ERFs at about 240 ms after stimulus onset. This result might depend on similar mechanisms as those related to P2 modulation in Lockwood and Tuomainen’s [23] study, that is, the load of sensory (auditory) information embedded in onomatopoetic word. There was no significant late-positivity effect as in the two mentioned studies in our data [23,28]; however, the interpretation of more effortful retrieval might as well be dependent on the use of ideophones, and the enhanced difficulty of making meta lexical decisions [28] is fairly task-specific.

### Clinical Applications

Possible clinical applications of the grounded cognition framework have been previously proposed [44]. It was proposed that patients with aphasia and lesions in motor areas could benefit from cognitive training with words that imply movement. This might add to conventional movement therapies and is supposed to induce neuroplasticity and regeneration in the affected areas. The effects of linguistic cognitive training on neural plasticity have been shown in healthy volunteers, thus delivering encouraging results [45]. First clinical tests have also been performed, but only as proofs of concepts and not in large cohorts of patients [46]. A similar cognitive improvement might be aimed at in patients with aphasia and lesions in auditory areas by applying linguistic training with sound-related words. The current ERFs results suggest that onomatopoetic verbs might suit such cognitive therapy programs.

## Supplementary Materials

The following supporting information can be downloaded at: https://www.mdpi.com/article/10.3390/brainsci12040481/s1, Table S1: Word stimuli. Figure S1: Grandaverage of power difference between onomatopoetic and non-onomatopoetic sound verbs across all channels.
