# Supplementary material for: Induced and Evoked Brain Activation Related to the Processing of Onomatopoetic Verbs
Source: Brain Sci. 2022 Apr 6;12(4):481. doi: 10.3390/brainsci12040481 (PMC9029984; doi:10.3390/brainsci12040481)
Supplement: Supplementary file 1 [file brainsci-12-00481-s001.zip › brainsci-1618360-supplementary.pdf]

Table S1: Word stimuli used in the experiment, sorted by group and with the related parameter values. Please note that not all onomatopoetic words in German are onomatopoetic when translated in English or in other languages.

**Onomatopoetic verbs**	**Onomatopoeia**	**Familiarity**	**Loudness**	**Length**	**Frequency**	**Question**	**Answer**	**Non-onomatopoetic verbs**	**Onomatopoeia**	**Familiarity**	**Loudness**	**Length**	**Frequency**	**Question**	**Answer**
ächzen (to groan)	2,9	3,4	2,4	6	16	1	No	ballern (to shoot)	2,2	3,6	3,7	7,0	16	2	No
donnern (to thunder)	2,8	3,9	3,8	7	15	2	No	beben (to quake)	2,1	3,7	3,2	5,0	16	1	No
gähnen (to yawn)	2,9	3,9	2,0	6	17	3	No	bersten (to break)	1,9	3,2	3,5	7,0	16	2	No
grölen (to bawl)	2,7	3,6	3,8	6	17	3	No	brodeln (to boil)	2,2	3,5	2,3	7,0	16	2	No
grummeln (to grumble)	2,9	3,7	2,0	8	19	1	No	erörtern (to discuss)	1,3	3,7	1,9	8,0	13	2	Yes
gurgeln (to gargle)	2,9	3,7	2,4	7	18	1	Yes	filtern (to filter)	1,4	3,6	1,4	7,0	15	1	Yes
hauchen (to breathe)	2,9	3,6	1,1	7	17	1	No	fluchen (to curse)	2,2	3,8	3,0	7,0	15	2	Yes
hupen (to honk)	2,7	3,9	3,7	5	16	2	No	fräsen (to mil-cut)	1,8	3,3	3,6	6,0	17	3	Yes
kichern (to giggle)	2,6	3,8	2,2	7	16	1	No	jammern (to whine)	2,0	3,9	2,7	7,0	13	3	No
klappern (to rattle)	2,7	3,8	2,9	8	16	1	No	kehren (to sweep)	1,6	3,7	2,0	6,0	12	2	No
klatschen (to clap)	2,9	3,9	3,4	9	14	1	No	küssen (to kiss)	1,6	3,9	1,3	6,0	13	3	No
klirren (to clink)	3,1	3,7	2,7	7	17	2	No	labern (to talk)	1,9	3,8	2,5	6,0	17	1	No

knattern (to rattle)	2,7	3,6	3,1	8	17	2	No	mahnen (to remind)	1,8	3,6	2,8	6,0	14	3	No
knistern (to crackle)	2,5	3,8	2,0	8	17	1	No	nieseln (to drizzle)	2,0	3,8	1,3	7,0	19	1	Yes
kreischen (to screech)	2,7	3,7	3,9	9	16	1	No	plaudern (to chat)	1,8	3,8	2,4	8,0	13	2	Yes
läuten (to ring)	2,5	3,7	3,3	6	14	1	No	plauschen (o chat)	1,6	2,9	2,2	9,0	18	2	Yes
mampfen (to munch)	2,8	3,5	2,2	7	19	2	Yes	raunen (to whisper)	2,2	3,4	2,1	6,0	18	3	No
murmeln (to mutter)	2,6	3,6	1,7	7	17	3	No	rieseln (to trickle	2,2	3,5	1,4	7,0	16	1	Yes
peitschen (to whip)	2,6	3,7	3,4	9	16	2	No	rinnen (to stream)	1,5	2,6	1,6	6,0	17	1	Yes
plätschern (to splash)	2,6	3,8	1,9	10	17	1	Yes	rotieren (to rotate)	1,6	3,6	2,2	8,0	15	2	No
prasseln (to patter)	2,8	3,6	2,9	8	17	2	No	schildern (to describe)	1,5	3,7	2,1	9,0	13	2	Yes
rappeln (to rattle)	2,7	3,4	2,7	7	18	2	No	schimpfen (to rant)	2,0	3,9	3,3	9,0	13	1	No
rascheln (to rustle)	2,5	3,8	1,8	8	17	2	No	schleifen (to grind)	2,2	3,7	2,9	9,0	14	2	No
rasseln (to clank)	2,8	3,6	2,6	7	17	2	No	schwatzen (to chatter)	1,9	3,7	2,5	9,0	17	2	Yes
rauschen (to sough)	2,9	3,8	2,2	8	15	2	No	sickern (to seep)	1,6	3,2	1,4	7,0	17	3	No
röcheln (to wheeze)	2,6	3,6	2,3	7	19	3	No	sieden (to simmer)	1,5	3,3	1,9	6,0	18	1	Yes
rülpsen (to burp)	2,7	3,7	2,9	7	18	3	No	spotten (to scoff)	2,2	3,5	2,8	7,0	16	3	No

schluchzen (to sob)	2,6	3,8	2,4	10	18	3	No	sprudeln (to bubble)	2,0	3,8	2,2	8,0	14	1	Yes
schlürfen (to slurp)	2,9	3,6	2,3	9	15	1	Yes	sprühen (to spray)	1,8	3,8	1,8	7,0	15	1	Yes
schreien (to scream)	2,5	3,9	3,9	8	12	2	Yes	stammeln (to stammer)	2,2	3,6	2,1	8,0	18	3	No
stöhnen (to moan)	2,8	3,8	2,7	7	15	1	No	tratschen (to gossip)	1,8	3,6	2,6	9,0	17	2	Yes
surren (to whir)	2,9	3,2	1,9	6	17	1	No	tröpfeln (to dripple)	2,2	3,7	1,6	8,0	18	1	Yes
ticken(to tick)	3,1	3,8	2,0	6	14	1	No	walzen (to mill)	1,9	3,0	2,9	6,0	18	1	No
zischen (to hiss)	3,1	3,7	2,2	7,0	16	1	Yes	zetern (to nag)	2,2	2,9	3,2	6,0	18	2	Yes
**averages**	2,8	3,7	2,6	7,4	16				1,9	3,5	2,4	7,2	16		

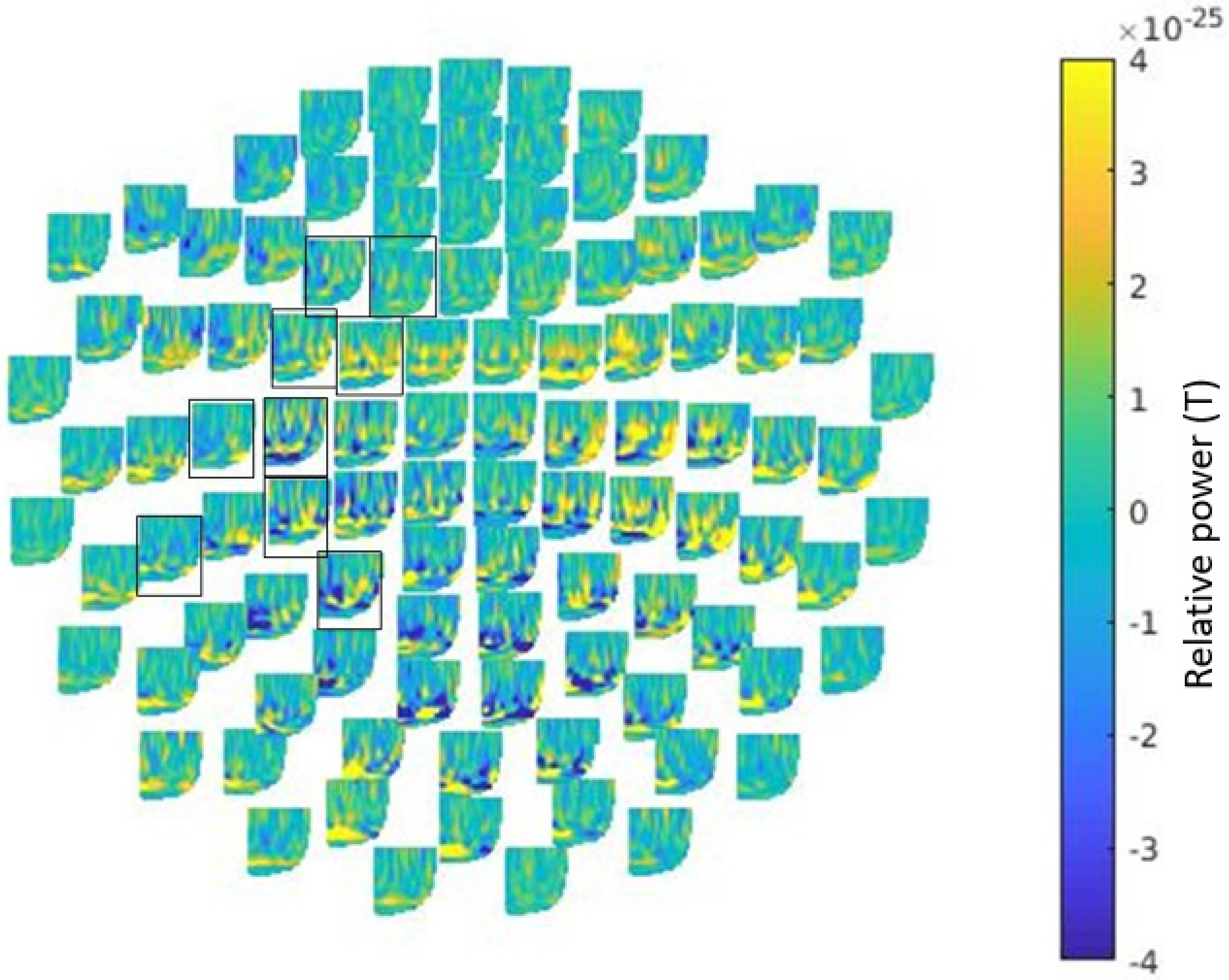

Figure S1: Grandaverage of power difference between onomatopoetic and non-onomatopoetic sound verbs across all channels. Each time-window starts 200 ms before word onset and ends 1000 ms after word onset. The frequency range is between 2 and 40 Hz. The black outline represents the channels selected for further analysis, taken from the localiser task in Niccolai et al. (2020).
